# The Utility of Supracutaneous Plating in the Staged Management of Compound Distal Femur Fracture: A Case Report

**DOI:** 10.7759/cureus.24659

**Published:** 2022-05-02

**Authors:** Aditya L Kekatpure, Khizar K Khan, Aashay L Kekatpure

**Affiliations:** 1 Orthopedic Surgery, Jawaharlal Nehru Medical College, Datta Meghe Institute of Medical Sciences (DMIMS), Wardha, IND

**Keywords:** osteomyelitis, cement spacer, extracutaneous plating, locking compression plate, induced membrane

## Abstract

The aim of this report is to draw attention to the use of a vilipend technique ‘supracutaneous plating’ in the management of compound distal femur fractures. Treatment of compound fractures of the distal femur with bone defects and microbial infection remains a challenging task for orthopaedic trauma surgeons. In this case report, we share our experience with the use of the locking distal femoral plate as an external fixator for the staged management of a compound infected distal femur fracture in a 22-year-old male patient. Staged procedures with proper planning give excellent results for infected fractures. Supracutaneous plating can be a viable and patient-friendly alternative in the staged management of compound distal femur fractures instead of the conventional external fixators.

## Introduction

Distal femoral fractures account for 6% of adult femoral fractures and 0.4% of all fractures [1,2]. High-velocity injuries lead to compound fractures and severe soft tissue damage. Also, pre-existing risk factors like diabetes mellitus and smoking contribute to a prolonged hospital stay, thereby increasing the risk of infection and non-union [3,4]. Treatment of compound fractures of the distal femur with bone defects and polymicrobial infection remains challenging for orthopaedic trauma surgeons. For limb salvage and early weight-bearing, long bones in the lower limbs require solid and rigid fixation [5]. Despite the developed hygienic standards, implants, and medications, the risk management and microbiological control strategies remain significant in increasing multi-resistant microbial trends [6].

By the conventional method, compound distal femur fractures are managed initially with wound debridement and external fixators in the form of circular or unilateral AO fixators, followed by definitive fixation after two to three weeks. The locking compression plates used as an extracutaneous plate are a better alternative to the conventional external fixator. Although having the same basic principles as a conventional external fixator, the advantages of extracutaneous plating are count worthy [7]. Complications like stiffness of the joint due to joint spanning, heavy weight of the rods, and pin site infections due to pin loosening can be avoided by using a locking compression plate as an external fixation device. Moreover, these are better accepted by the patients as they are less bulky.

The diamond concept (Masquelet technique) is an alternate method to the current Ilizarov technique of excision followed by the distraction osteogenesis procedure. However, the difficulties faced, like pin loosening, pin site infection, and joint stiffness, are common with Ilizarov’s technique [3,8,9].

Here, we report a case of infected distal femur fracture, managed by an antibiotic-loaded cement spacer and extracutaneous plating, followed by definitive fixation and allogenic bone grafting. The outcome obtained using this method was excellent and worth reporting.

## Case presentation

**Figure 1:**
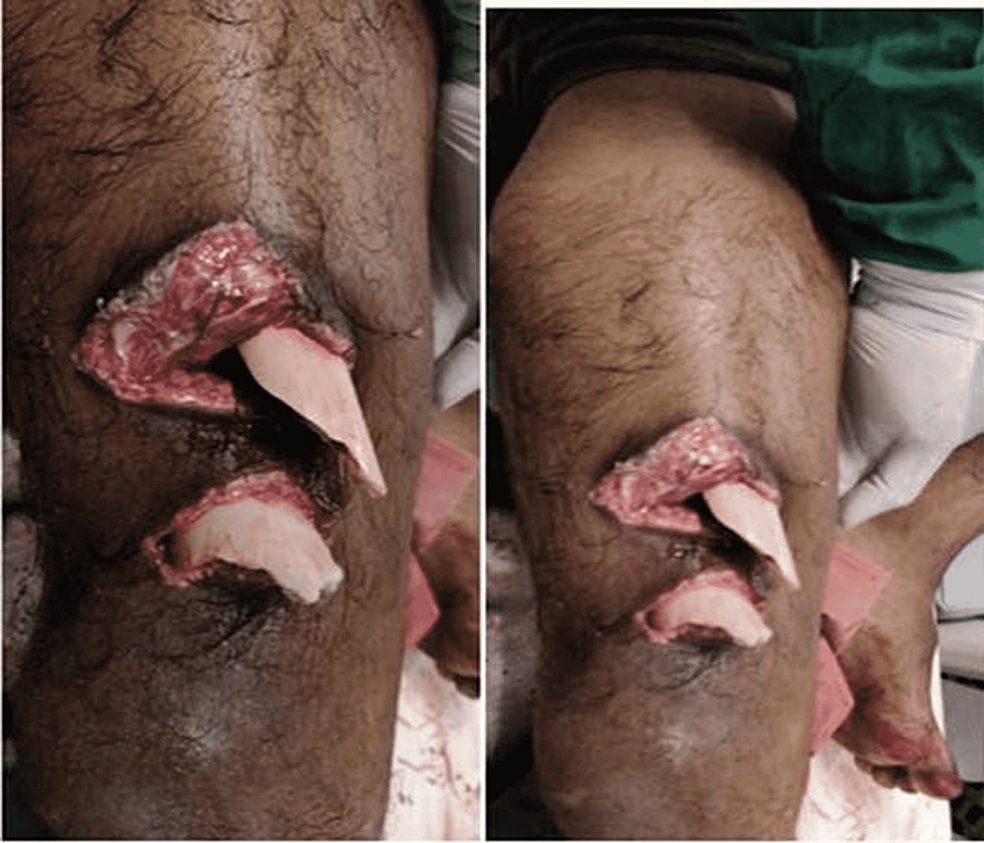
Clinical image of the wound at the time of presentation

**Figure 2:**
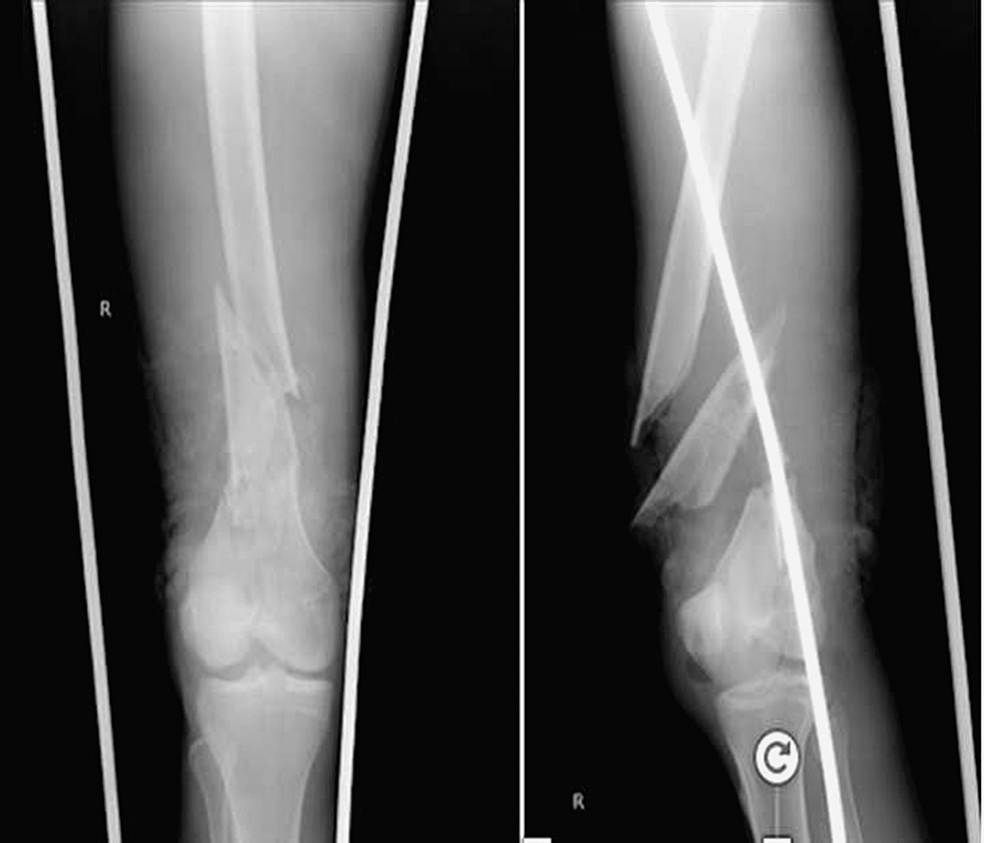
X-ray right femur showing segmental supracondylar femur fracture

A 22-year-old male patient came to the casualty with an alleged history of a road traffic accident, sustaining an injury to his right lower limb. At the time of presentation, the patient had a segmental compound supracondylar femur fracture on the right side with two spikes of bone protruding out of the right thigh. Through lavage with betadine, hydrogen peroxide and normal saline were given to the wounds. The patient also had pain and swelling over the right leg with a crushed foot injury with multiple visible bone fragments and ruptured tendons over its dorsal aspect. The limb was stabilized on a Thomas splint, and all the bone fragments were pushed inside the skin. After hemodynamic stabilization with fluid resuscitation and two whole blood transfusions, a series of X-rays were done and the patient was diagnosed with compound grade IIIB segmental supracondylar fracture of the femur on the right side (Figures 1-2), compound grade II fracture of the midshaft tibia on the right side, and compound grade IIIB 2nd-5th metatarsal fracture on the right side.

**Figure 3:**
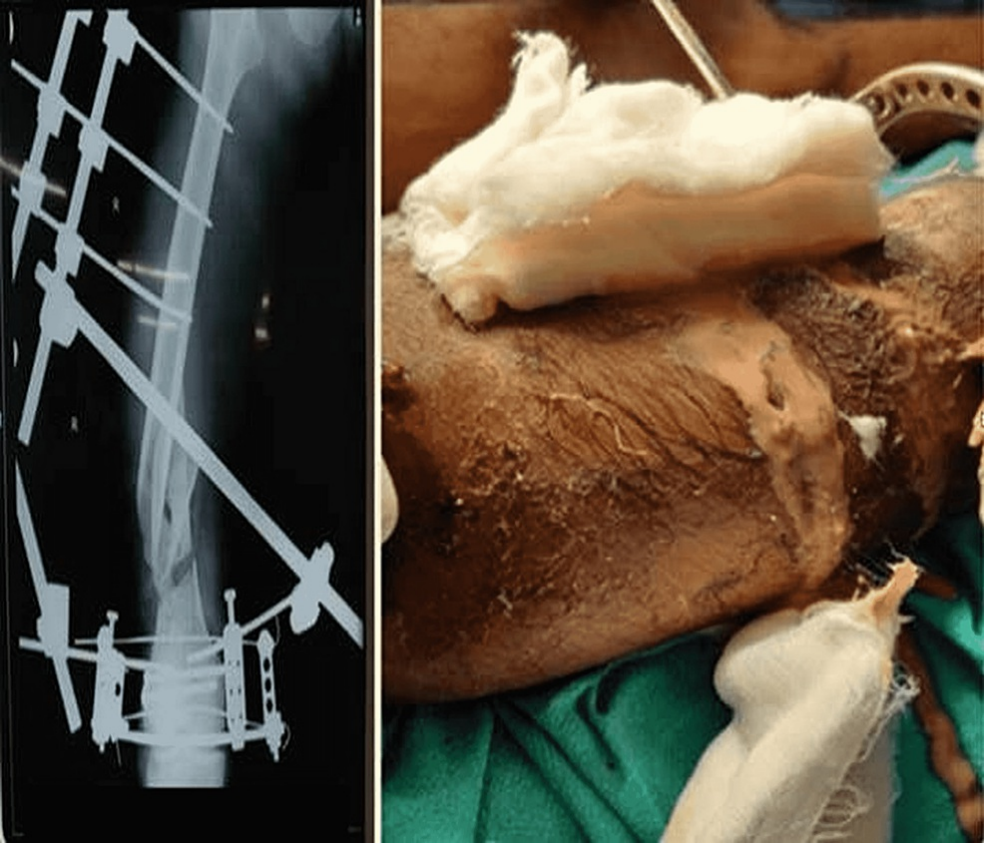
X-ray right femur showing external fixator over distal femur and clinical image of pouring pus after infection

As the patient was COVID positive, he was then surgically managed in an isolated operation room. Thorough irrigation with thorough wound debridement and external fixator application for supracondylar fracture femur, intramedullary nailing for fracture mid-shaft tibia, and closed reduction with a k-wire fixation for metatarsal fractures. The segmental bone piece of the femur was kept in place and stay suturing was done. A broad-spectrum antibiotic, third-generation cephalosporin (which has Gram-positive and extended Gram-negative coverage), was started. Later, the wound over the thigh got infected with the frank purulent discharge present on the 10th day (Figure 3). The pus was sent for culture and antibiotic sensitivity testing, which showed growth of *Pseudomonas aeruginosa* on one occasion and coagulase-negative staphylococci on another occasion. Organisms were sensitive to gentamicin, amikacin, and clindamycin, which were started accordingly.

**Figure 4:**
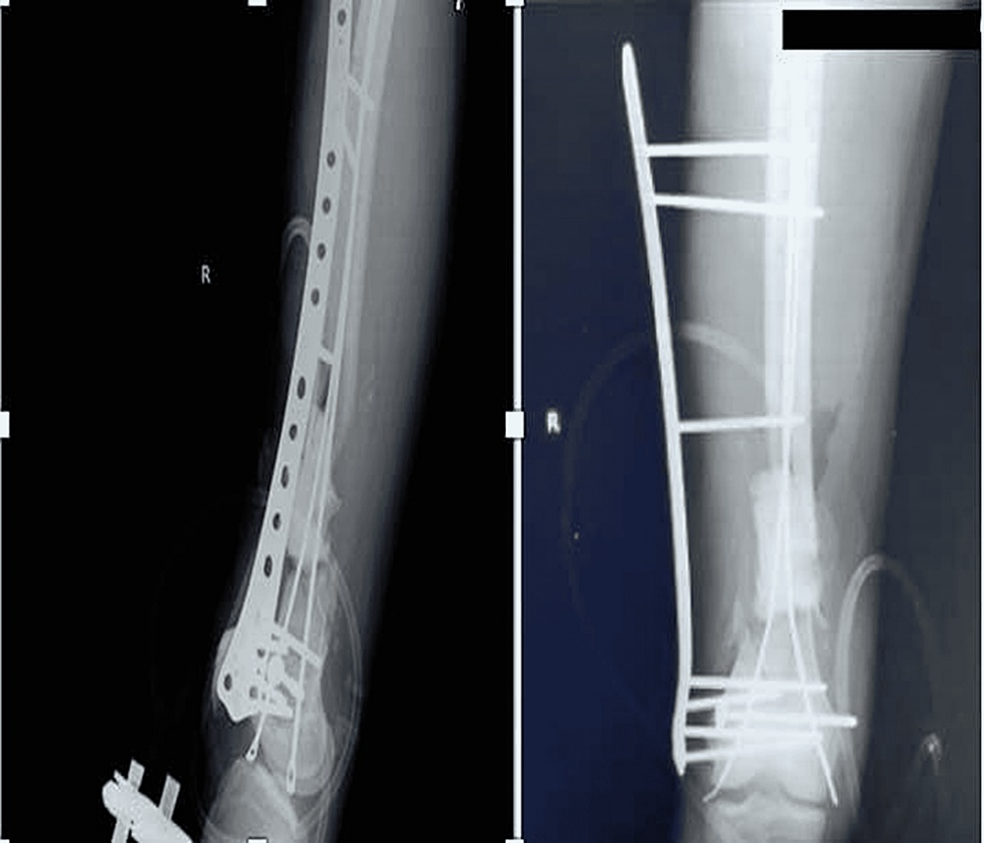
X-ray of right femur showing extracutaneous plate with cement spacer

**Figure 5:**
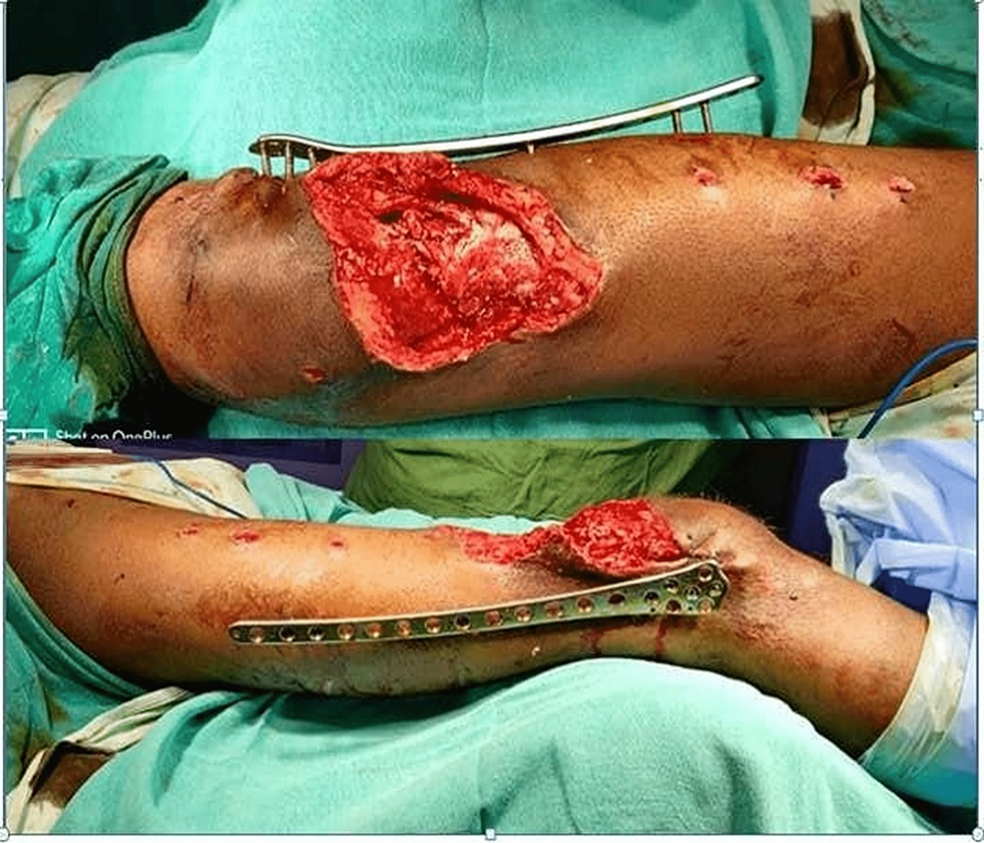
Post-operative clinical image of right thigh with extracutaneous plate

In view of continued pus discharge, the external fixator was removed from the femur, the devitalized segment of the femur was removed, and thorough debridement was done. In addition, two ender nails were inserted (ender nails are solid nails that can be used in compound fractures and provide additional stability to the construct and the cement spacer as well), a 2 g vancomycin impregnated polymethylmethacrylate cement spacer was kept in the bone gap, and extracutaneous plating was done using a 13-hole distal femur plate (Figures 4-5). Intravenous antibiotics as per the sensitivity were continued for 15 days, followed by oral antibiotics. ESR and CRP readings were significantly improving along with the clinical improvement. Wound management was done by two sittings of vacuum-assisted closure (VAC), followed by skin grafting on healthy granulation tissue.

**Figure 6:**
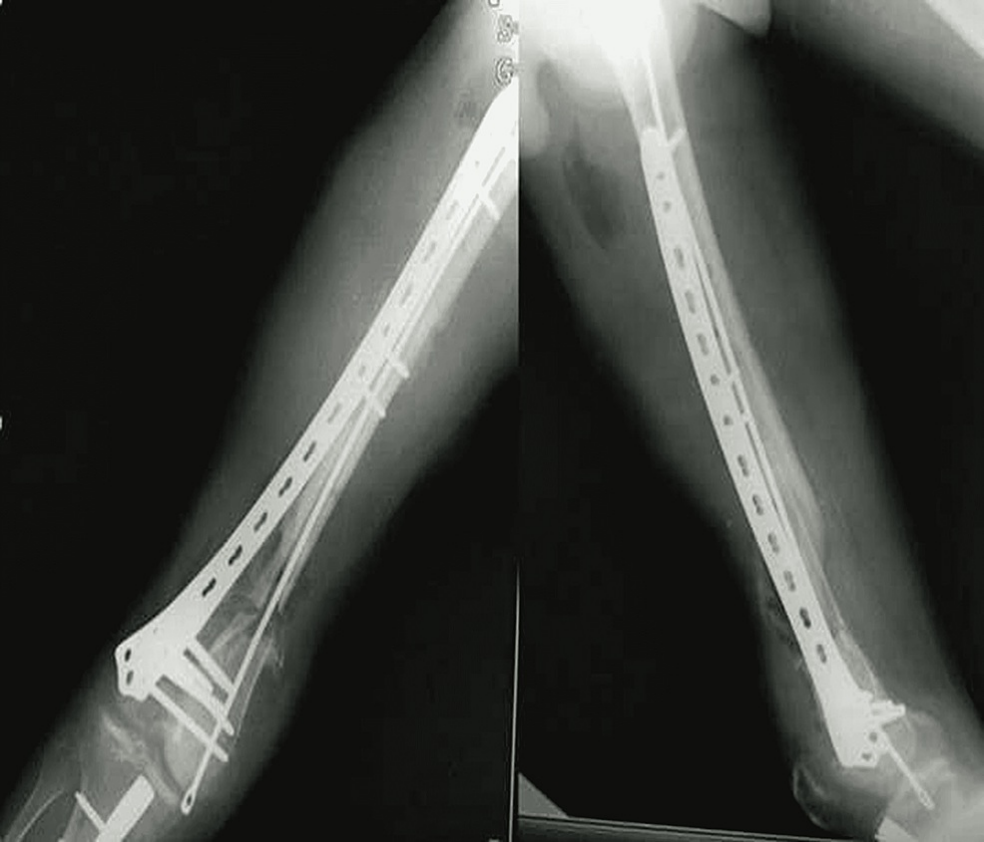
X-ray right femur showing internal fixation with distal femur locking plate and a medially placed ender nail in situ

After eight weeks, the wound was properly healed with no signs of infection. Therefore, definitive fixation was planned and the extracutaneous plate, along with the ender's nail, and bone cement, was removed. An induced membrane was seen over the spacer, which was preserved, and an autograft using a split fibula graft was placed inside the membrane, filling the bone gap. Rigid fixation was done laterally using a 13-hole distal locking femur plate, and an ender's nail was inserted medially to prevent medial collapse (Figure 6).

**Figure 7:**
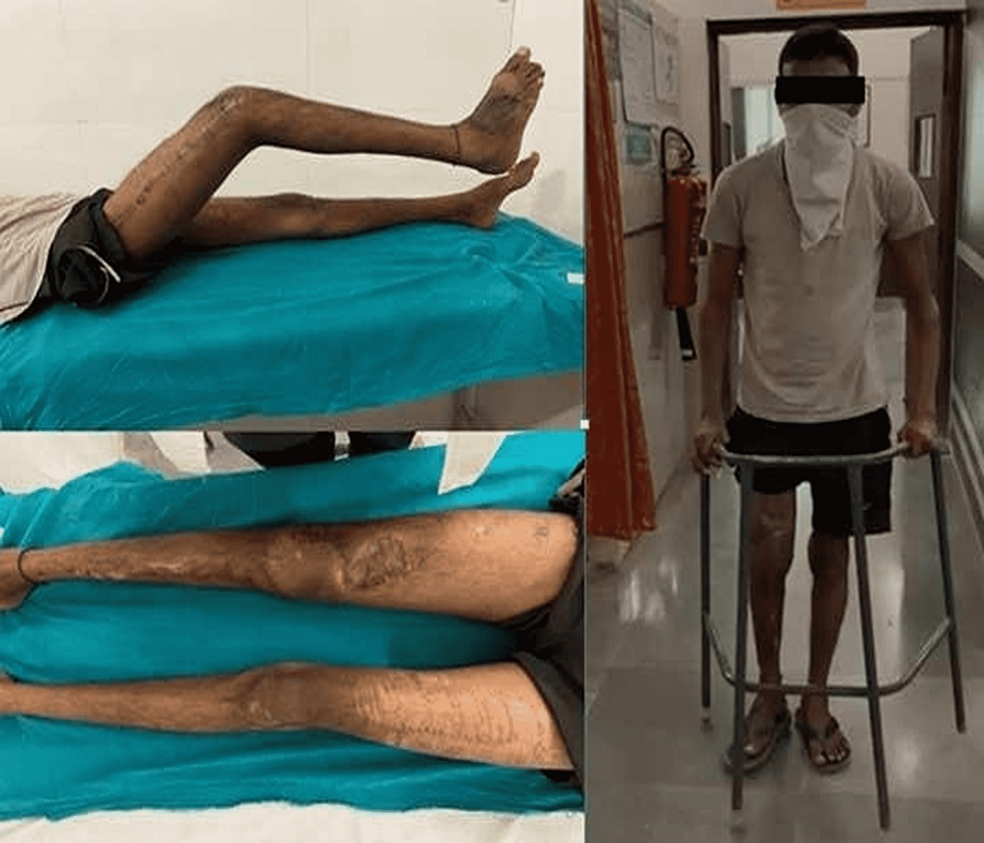
Clinical image showing healed wounds and good clinical outcome

The patient was allowed assisted weight-bearing after 12 days (i.e., after the surgical wound healed and suture removal was done). Closed chain exercises of the quadriceps and hamstrings were started on the very next day of the procedure, along with dynamic ankle pump exercises. Gentle mobilization of the knee was started after suture removal. The knee range of movement gained was 0 to 90 degrees. There were no signs or symptoms of infection, even after one year of follow-up (Figure 7).

## Discussion

Infection rates of 11% to 22% are noted with the treatment of compound fractures of long bones [10]. Such an infected non-union requires radical debridement along with the removal of dead bone as well as soft tissue, which leads to long defects [11]. Despite various modalities for the reconstruction of these types of defects after resolution of infection and recovery of soft tissue, none of them is the gold standard, and the choice of management depends on the preference of the surgeon [5].

Extracutaneous plating in the management of compound fractures has gained attention in the past decade as it played a key role in addressing many avoidable complications. However, the stability of the locking compression plate is less than the stability of traditional external fixators, and theoretically, it does provide optimum stability at the fracture site [8]. But the advantages outweigh them in many domains. Stability can be increased many folds by using a contoured locking plate with multiple distal locking screw holes. This is more stable as compared to the two Schanz pins used in a conventional external fixator. External locking plates are more patient-friendly as the patient can wear clothes over the frame and also travel without any inconvenience. Stiffness of the joint due to the heavyweight of the external fixator is less likely to occur as the weight of the compression plate is comparatively light. Removal of this implant can be done as a minor procedure without anaesthesia or under local anaesthesia. Assessment of the healing is better appreciated on an X-ray due to less hindrance by the implant [12-14].

Conventional fixators can be reinforced by adding connecting rods, but the external attachment of a compression plate cannot be done. Although there is less scope for realignment with extracutaneous plates, dynamization can be done by the removal of screws adjacent to the fracture site.

The technique of membrane induction is an established modality of treatment for the reconstruction of large bone defects caused by the traumatic loss of bone, osteomyelitis, or tumour excision [15]. To decrease difficulties and increase efficacy, it is necessary to understand the technique's fundamental concepts. The membrane induced around the polymethylmethacrylate spacer acts as a chamber for bone graft and facilitates regeneration. The pros of this technique are: (i) the membrane that is induced keeps the graft in its place and helps in revascularisation, consolidation, and bone formation; (ii) after enough meticulous debridement, a cancellous graft can be used even in cases of infection or malignancy; (iii) anatomy is preserved; and (iv) augmentation of the graft can be done as per the requirements. This technique requires two-staged procedures, which are eventually unavoidable with any modality.

The most widely used modalities for the reconstruction of long bone defects are free fibular grafting, non-vascularized fibular grafting, and distraction osteogenesis using Ilizarov’s ring fixator. Although these procedures are considered advantageous, they have inherent shortcomings. Free fibular grafting requires demanding microsurgical techniques and thereby prolongs surgical time. The risk of inadequate acceptance of non-vascularized fibular grafts or fracture of the fibular graft cannot be excluded [16]. When performing callus transfer using Ilizarov’s ring fixator, there is an increased risk of infection when in proximity to the joint. The amount of time required for the distraction following consolidation and the continuous active manual distraction requires high patient compliance. Ilizarov’s fixator gives excellent stability for the demands of physiologic limb movement in the tibia, but the soft tissue problems are higher with femur and knee range of motion (ROM) being restricted [9,17]. Irrespective of any method used for reconstruction, the patient needs to be informed regarding the longevity, difficulty, and requirement of further procedures. Considering the merits and demerits of these procedures, alternative methods can be used in certain cases.

## Conclusions

Using a locking compression plate as an external fixator device is a very good alternative to conventional external fixation devices in the management of compound injuries of the lower limb. Although this technique has been known to trauma surgeons for over a decade, it has been of little to no use in clinical practice as the availability of data in the literature regarding its advantages and outcomes is scarce. This particular technique should be in utilization because it has the advantages of being patient-friendly in various ways, like the ability to wear trousers over the extracutaneous plate, the easiness of using public transport, allowing early rehabilitation, and it is less cumbersome. It facilitates the surgeon to construct a rigid fixation in juxta-articular fractures by allowing the surgeon to insert multiple screws in the distal fragment, and it also provides space to address wound management procedures like VAC application, skin grafting, and flap transfers.
